# Increase in serum and salivary neutrophil gelatinase-associated lipocalin levels with increased periodontal inflammation

**DOI:** 10.1590/1678-7757-2020-0276

**Published:** 2020-09-28

**Authors:** Aykut Tan, Nilgün Gürbüz, Furkan İlker Özbalci, Özgür Koşkan, Zuhal Yetkin Ay

**Affiliations:** 1 Süleyman Demirel University Faculty of Dentistry Department of Periodontology Isparta Turkey Süleyman Demirel University, Faculty of Dentistry, Department of Periodontology, Isparta, Turkey.; 2 Süleyman Demirel University Faculty of Medicine Department of Medical Biology Isparta Turkey Süleyman Demirel University, Faculty of Medicine, Department of Medical Biology, Isparta, Turkey.; 3 Isparta University of Applied Science Faculty of Agriculture Department of Biometrics Isparta Turkey Isparta University of Applied Science, Faculty of Agriculture, Department of Biometrics, Isparta, Turkey.

**Keywords:** Gingivitis, Innate immunity, Inflammation, Periodontitis

## Abstract

**Objective:**

This study aimed to determine serum and salivary levels of neutrophil gelatinase-associated lipocalin (NGAL) and evaluate NGAL correlation with key anti-interleukin 10 (IL-10) and pro-inflammatory (IL-1β) cytokines in different severities of periodontal diseases. We also calculated the systemic inflammation using the periodontal inflamed surface area (PISA) to evaluate its correlation with NGAL in the study groups.

**Methodology:**

Eighty systemically healthy and non-smoking individuals were separated into four groups of 20: clinically healthy (Group 1), gingivitis (Group 2), stage I generalized periodontitis (Group 3, Grade A), and stage III generalized periodontitis (Group 4, Grade A). Sociodemographic characteristics and periodontal parameters were recorded, and PISA was calculated. The serum and salivary levels of interleukin (IL)-1β, IL-10, and NGAL were determined using the enzyme-linked immunosorbent assay (ELISA).

**Results:**

We observed a significant increase in serum and salivary NGAL levels from healthy to periodontitis groups (p=0.000). Group 2 presented significantly higher serum and salivary IL-10 levels and salivary IL-1β levels than Group 3 (p=0.000). Serum and salivary parameters (IL-1β, IL-10, and NGAL levels) were strongly positively correlated to periodontal parameters and PISA values (p=0.000). Groups 2 and 3 showed overlapping PISA values.

**Conclusion:**

The overlapping PISA values found in Groups 2 and 3 suggest that gingivitis might progress to a systemic inflammatory burden somewhat comparable to stage I periodontitis. This finding is supported by the higher serum and salivary cytokines/mediators levels in the gingivitis group than in stage I periodontitis group. Serum and salivary NGAL levels increased proportionally to disease severity and PISA. NGAL seems to play a role in the pathogenesis of periodontal disease, within the limitation of our study.

## Introduction

Periodontitis is a chronic inflammatory disease that shares common mechanistic pathways with other systemic inflammatory diseases. Periodontal inflammation is regarded as a risk factor for systemic inflammation and disease at distant tissues.^1^ Studies have suggested several possible mechanisms by which periodontitis might affect distant organs: the first is direct migration and colonization of periodontal microbial species to distant areas, resulting in an inflammatory reaction distant from the invasion point; the second is a systemic inflammation resulting from metastatic periodontal inflammation or activated soluble inflammatory pathways by periodontal bacteria.^1^^,^^2^ Nesse, et al.^3^ (2008) state that the amount of inflamed periodontal tissue determines the systemic inflammatory burden. The authors developed a meter that compares the bleeding pocket epithelium length with the periodontal inflamed surface area (PISA).^3^

Various studies used the PISA to understand the association between systemic diseases and periodontitis.^4^^-^^6^ Considering their results, PISA may be used as a continuous periodontal variable in conjunction with microbiological or biochemical variables (cytokine(s)/mediator(s)) in periodontal medicine studies, to clarify any potential role in the pathogenesis of both periodontal and systemic diseases.

In periodontal disease, host inflammatory cells are mobilized on inflammation site and inflammatory cytokines are released from fibroblasts, macrophages, connective tissue, and junctional epithelial cells.^7^ One of the most important cytokines in the periodontitis-related immune response and tissue destruction is interleukin (IL)-1β, which is produced by macrophages, monocytes, lymphocytes, epithelial cells, fibroblasts, and osteoblasts,^8^ has a potent pro-inflammatory function,^8^ and is reported to be present in increased levels in the serum, saliva, and gingival crevicular fluid (GCF) of periodontitis patients when compared to controls.^9^^-^^11^ Studies reported a decrease in serum and GCF IL-1β levels after non-surgical periodontal treatment.^11^^,^^12^ To control immune-inflammatory response and prevent tissue damage, regulatory T cells produce cytokines, such as IL-10, to inhibit the synthesis of pro-inflammatory cytokines and suppress immune-inflammatory-driven alveolar bone resorption.^13^

Neutrophil gelatinase-associated lipocalin (NGAL) belongs to the lipocalin family and has the ability to bind iron, fatty acids, prostaglandins, steroids, and matrix metalloproteinases.^14^ A study reported NGAL to play an important role in mediating innate immunity response to bacterial infections^15^ by sequestrating iron-loaded siderophores. It is also a neutrophils chemoattractant, promoting their maturation, adhesion and extravasation, and their phagocyte capacity, besides activating regulatory T cells.^14^ Due to the elevated circulating NGAL levels in the early stages of inflammation, it is considered a risk indicator for nephropathy, kidney dysfunction, and liver homeostasis.^16^ IL-1β was found to upregulate NGAL in human epithelial cells^16^ and to possibly comprise a determinant for circulating NGAL in systemic inflammations.^17^ A study found IL-10 overexpression by primary macrophages to enhance their pro-resolution activity in complex inflammation-associated pathologies.^18^ Likewise, blocking of NGAL production in macrophages reduced IL-10 protective effects in a kidney ischemia/reperfusion injury model, substantiating NGAL-associated pro-proliferative and anti-inflammatory properties.^18^

Although NGAL role in cases of kidney dysfunction was widely investigated, studies on its function in the pathogenesis of periodontal disease and its association with serum and salivary levels and key cytokines in periodontitis are still scarce. The literature reported NGAL presence in GCF and saliva^18^^,^^19^ and suggested the neutrophils extravasated is main source of this protein in these biological fluids and in gingiva.^19^ NGAL has been reported to be a salivary biomarker in patient monitoring and disease activity control,^20^ and urinary NGAL has been indicated for clinicians to screen periodontitis.^21^ Considering the lack of studies in the existing literature on periodontitis regarding the role of NGAL and its association with cytokines/mediators in the pathogenesis of periodontal diseases, it is logical to evaluate NGAL association with IL-1β and IL-10 and determining its function in the pathogenesis of periodontitis.

This study aimed to evaluate serum and salivary NGAL levels in different types of periodontal diseases and different levels of disease severity. We also sought to investigate whether NGAL levels in local (saliva) and systemic environments (serum) proportionally increase with increased severity, bleeding, and size of the ulcerated periodontal inflamed surface area (PISA).

## Methodology

This study was approved by the Clinical Research Ethics Committee of the Faculty of Medicine of the Süleyman Demirel University (SDU) (16.08.2017, decision No. 121) and conducted in accordance with the Declaration of Helsinki from 1975 as revised in 2013. The study population consisted of 80 participants who applied to the SDU, Faculty of Dentistry, Department of Periodontology, between September 2017 and November 2018. All participants were required to sign a consent form before participating in the study.

### Exclusion criteria

Individuals were excluded from the study if they: were previously diagnosed with a systemic disease, older than 65, using any medication affecting values of periodontal parameters, smokers, had received periodontal or antibiotic treatment within the six months prior to the study, presented necrotizing ulcerative gingivitis, were receiving orthodontic treatment, had alcohol or substance dependence, had less than 20 teeth, or refused to sign the consent form.

### Patient groups

Patients were assessed for evidence of periodontal disease and classified according to the New Classification Scheme for Periodontal and Peri-Implant Diseases and Conditions proposed in the 2017 World Workshop^22^ and divided into four groups: periodontally healthy (Group 1, n=20), gingivitis (Group 2, n=20), stage I generalized periodontitis (Group 3, n=20), and stage III generalized periodontitis (Group 4, n=20). All patients with periodontitis (Group 3 and Group 4) were Grade A (no attachment loss over 5 years, % bone loss/age: 0.25-1.0 mm, heavy biofilm deposits with low levels of destruction, non-smoker, normoglycemic/no diagnosis of diabetes).^22^

Periodontally healthy group is characterized by no or minimal levels of clinical inflammation in a periodontium with normal support (clinical periodontal health: no/minimal bleeding on probing [BOP <10%], normal gingival sulcus depth and bone heights, controlled modifying and predisposing factors).^23^ Gingivitis group is characterized by no probing attachment loss (AL), and radiographic bone loss and BOP score ≥ 10% and ≤ 30% (gingivitis in an intact periodontium).^23^ Stage I periodontitis is characterized by 1 to 2 mm AL at greatest loss site, radiographic bone loss in coronal third (<15%), and no tooth loss due to periodontitis.^22^ Stage III periodontitis is characterized by ≥ 5 mm AL at greatest loss site, radiographic bone loss extending to mid-third or apical third of root, and ≤ 4 teeth loss due to periodontitis.^22^ Periodontitis extent was determined as generalized (≤ 30% of teeth involved) according to the criteria proposed by the 1999 International Workshop for the Classification of Periodontal Diseases and Conditions and by the 2017 World Workshop on the Classification of Periodontal and Peri-Implant Diseases and Conditions.^22^^,^^23^

Patients were also evaluated for healthy, suffering from gingivitis, and suffering from periodontitis – criteria proposed by the 1999 International Workshop for the Classification of Periodontal Diseases and Conditions.^24^ Classification was further sub-divided by severity (mild, moderate, or severe), as jointly defined by the Centers for Disease Control and Prevention (CDC) and the American Academy of Periodontology (AAP).^25^ Considering CDC-AAP classification,^25^ groups may be summarized into: periodontally healthy (Group 1), gingivitis (Group 2), mild periodontitis (Group 3, ≥ 2 interproximal sites with AL ≥ 3 mm, and ≥ 2 interproximal sites with probing depth (PD) ≥ 4 mm [on different teeth] or one site with PD ≥ 5 mm), moderate and severe periodontitis (Group 4, moderate: ≥ 2 interproximal sites with AL ≥ 4 mm [on different teeth], or ≥ 2 interproximal sites with PD ≥5 mm [on different teeth]; and severe: ≥ 2 interproximal sites with AL ≥ 6 mm [on different teeth] and ≥ 1 interproximal site with PD ≥ 5 mm).

### Sample size determination

Sample size was determined according to the metrics established by Leira, et al.^26^ (2018), at 0.05 significance level and 80% statistical power. Considering PISA value between periodontally healthy patients and those with severe periodontitis, we found a minimum of 20 subjects per group.

### Sociodemographic and dental characteristics

Sociodemographic (age, gender, education level, monthly income, height, and weight) and dental characteristics (toothbrushing frequency, frequency of dental visits, use of interdental hygiene equipment, such dental floss, waterpick, toothpick) were recorded.

### Periodontal evaluation

A single qualified investigator (A. Tan) performed periodontal examination (intraclass correlation coefficients [ICC] for periodontal probing depth [PPD] was 0.997 and for AL 0.995). Gingival index (GI)^27^ and plaque index (PI)^28^ were recorded for four sites on each tooth (mesiobuccal, midbuccal, distobuccal, and mesiolingual/palatal). BOP, AL and PPD scores were obtained for six sites (mesiobuccal, mid-buccal, distobuccal, mesiolingual, mid-lingual, and distolingual) using a Williams periodontal probe (Hu-Friedy Manufacturing Corp., Chicago, IL, USA.). All measurements were rounded to the nearest 0.5 mm, except for PPD and AL, which were rounded to the nearest whole mm. BOP percentage was calculated as the number of teeth exhibiting any bleeding.^29^ BOP scores obtained from the six sites were used for calculating periodontal inflamed surface area (PISA) and periodontal epithelial surface area (PESA) scores.^3^ For each tooth, the PESA was calculated using the clinical AL and recession area and the PISA was obtained from PESA multiplied by the number of sites with BOP. The sum of individual PISA values results in the full-mouth PISA value, in square mm^3^.

### Saliva samples

Unstimulated whole saliva was collected from all patients using a passive drool into sterile plastic tubes between 9 and 10 am, for 5 minutes, according to the method established by Navazesh and Kumar^30^ (2008). Participants were requested to refrain from eating, drinking, chewing gum, or breath mints, as well as performing oral hygiene procedures for at least one hour prior to saliva collection. After collection, samples were placed on ice and aliquoted before being storaged at −80°C.

### Serum samples

Fasting blood samples from the antecubital vein were collected using blood collection tubes with anticoagulant (EDTA, Greiner AG, Kremsmünster, Austria) and centrifuged at 3000 × g for 10 minutes. Before analysis, serum samples were portioned and stored at −80°C.

After all the periodontal measurements were recorded and salivary and serum samples were taken, periodontal therapy was administered according to the individual needs.

### Biochemical analysis

Serum and saliva IL-1β, IL-10, and NGAL levels were determined using human-specific enzyme-linked immunosorbent assay (ELISA) kits (Shangai YLbiont Biotech Co., Shangai, China), which is based on the biotin double-antibody sandwich technology to quantify specific IL-1β, IL-10, and NGAL antigens. Serum samples were diluted 1:10 and saliva 1:50 before analysis, assuming the specific standard curve with eight different concentrations for each parameter. Kits' sensitivity values were 10.07 pg/L (IL-1β), 12.3 pg/L (IL-10), and 5.01×10^6^ pg/L (NGAL).

### Statistical analysis

Data were analyzed to verify whether they fulfilled parametric tests preconditions (Box' M test for homogeneity of variance-covariance matrices and Kolmogorov-Smirnov test for normal distribution prerequisites). If preconditions were fulfilled, data were analyzed using one-way analysis of variance (ANOVA); if not, Kruskal-Wallis test was applied. In the parametric case, Tukey's multiple comparison test was used after ANOVA to determine statistical differences among groups. In the non-parametric case, the Bonferroni-Dunn test was applied after the Kruskal-Wallis test to identify multiple comparisons. Categorical demographic data (gender, education level, monthly income, dental visit frequency, brushing frequency, and interdental cleaning frequency) was analyzed with the chi-square test. Spearman's correlation was used to determine any correlations between parameters. A p-value less than or equal to 0.05 was considered statistically significant. All statistical analyses were performed with commercial statistical software (IBM SPSS Statistics for Windows, version 23, IBM Corp., Armonk, NY, USA).

## Results

Eighty patients (35 females and 45 male) aged between 25 and 63 years participated in the study. Table 1 shows the study population sociodemographic parameters and characteristics. We found no statistically significant differences between Groups 3 and 4 (p=0.966) and Groups 1 and 2 (p=0.142) regarding age. Mean age was significantly lower in Group 1 than in Groups 3 and 4 (Groups 1 and 3, p=0.001, Groups 1 and 4, p=0.000), but Group 2 showed no significant difference in relation to other groups (Group 1, p=0.142; Group 3, p=0.330; Group 4, p=0.142). We found a strong negative correlation between periodontitis severity and education level (p=0.000). Monthly income showed no significant difference among groups (p=0.880). As for body mass index (BMI), we observed both statistically significant (Groups 1 and 4, p=0.031) and non-significant differences (Group 1 and 2, p=0.254; Group 1 and 3, p=0.076; Group 2 and 3, p=0.934; Group 2 and 4, p=0.774; Group 3 and 4, p=0.983).

**Table 1:** Sociodemographic parameters and characteristics of the study groups

		Groups			
Parameters	Categorizations	Group 1	Group 2	Group 3	Group 4
Age	Min.-Max.	25-50	25-54	25-60	25-63
	Mean±SD	29.00±5.56§, ‖	35.05±8.52	39.80±10.61†	41.10±9.77†
Gender (n)	Female	7	10	14	9
Education level (n)	Elementary (8 years)	1	3	8	8
	Secondary (8-12 years)	0	5	6	7
	Higher (12 years<)	19	12	6	5
Monthly income (n)	0-1500 TL	2	4	2	0
	1500-3000 TL	6	7	10	11
	>3000 TL	12	9	8	9
BMI (kg/m2)	Min.-Max.	18.4-27.5	18.8-28.7	18.8-32.9	19.7-34.9
	Mean±SD	22.64±2.66‖	24.59±2.98	25.21± 3.51	25.59±3.93†
Dental visit frequency (n)	In case of need	0	1	17	20
	1/ 2-3 years	0	4	1	0
	1 or 2 times/year	20	15	2	0
Brushing frequency (n)	Rarely	0	1	1	1
	2-3 times/week	0	4	6	10
	1-2 times/day	20	15	13	8
Interdental cleaning (n)	Never	3	18	18	20
	Rarely	4	1	2	0
	2-3 times/week	7	1	0	0
	1-2 time/day	6	0	0	0

NOTES: 1 USA Dollar currency rate was between 3.66 and 5.24 TL and the minimum wage was 1500 TL during the period within which this study was conducted. BMI: body mass index.

† significantly different than Group 1,

‡ significantly different than Group 2,

§ significantly different than Group 3,

‖ significantly different than Group 4 (p<0.05, Chi-Square test, ANOVA, and Tukey's Test.).

Table 2 shows PISA and PESA values for the four groups. Periodontal inflamed surface area (PISA) and periodontal ephithelial surface area (PESA) values increased with disease severity and were significantly different among groups (p=0.000). Group 4 presented the highest plaque index (PI) and gingival index (GI) values and Group 1 the lowest. We found no statistically significant differences in PI and GI values between Groups 2 and 3 (p>0.05) and in periodontal parameters, (periodontal probing depth -PPD, attachment loss – AL, PISA, and PESA) among all groups (p<0.05).

**Table 2:** Dental and periodontal characteristics and periodontal epithelial and periodontal inflamed surface area (PISA and PESA) values of the study population

		Groups			
Parameters		Group 1	Group 2	Group 3	Group 4
NT	Mean Rank	51.45§, ‖	44.03§, ‖	28.9 3†, ‡	37.60†, ‡
	Min.-Max.	24-28	23-28	20-28	24-28
	Mean±SD	27.70±0.98	27.20±1.44	25.35±2.89	26.90±1.41
PI	Mean Rank	10.50‡, §, ‖	40.45†, ‖	47.38†, ‖	63.68†, ‡, §
	Min.-Max.	0.25-0.88	1.01-2.54	1.60-2.13	1.81-2.91
	Mean±SD	0.38±0.16	1.80±0.36	1.93±0.15	2.20±0.26
GI	Mean Rank	10.50‡, §, ‖	39.63†, ‖	45.78†, ‖	66.10†, ‡, §,
	Min.-Max.	0.13-0.73	1.30-2.25	1.66-2.04	1.86-2.70
	Mean±SD	0.33±0.13	1.77±0.25	1.90±0.11	2.15±0.20
BOP (%)	Mean Rank	10.50‡, §, ‖	45.98†, §, ‖	52.03†, ‡	53.50†, ‡
	Min.-Max.	0-8	85-100	93-100	100-100
	Mean±SD	6±3	97±5	99±2	100±0
PPD (mm)	Mean Rank	10.55‡, §, ‖	31.20†, §, ‖	50.90†, ‡, ‖	69.35†, ‡, §
	Min.-Max.	1.08-1.72	1.67-2.48	2.04-3.05	2.75-6.16
	Mean±SD	1.34±0.17	2.09±0.20	2.72±0.26	3.80±0.89
AL (mm)	Mean Rank	10.55§, ‖	30.5 5 §, ‖	51.50†, ‡, ‖	69.40†, ‡, §
	Min.-Max.	0	0	2.43-3.75	3.11-7.85
	Mean±SD	0	0	3.09±0.41	4.61±1.35
PISA (mm^2^)	Mean Rank	10.50‡, §, ‖	31.90†, §, ‖	49.55†, ‡, ‖	70.05†, ‡, §
	Min.-Max.	0-34.51	649.78-1191.61	962.57-1467.79	1250.77-3353.57
	Mean±SD	14.89±9.30	893.38±158.04	1187.67±132.52	2152.41±649.09
PESA (mm^2^)	Mean Rank	10.7‡, §, ‖	31.65†, §, ‖	49.9†, ‡, ‖	69.75†, ‡, §
	Min.-Max.	585.46-839.65	832.99-1297.89	986.32-1694.88	1342.35-3934.64
	Mean±SD	754.39±30.89	1057.18±117.66	1352.84±169.96	2313.39±721.11

† significantly different than Group 1,

‡ significantly different than Group 2,

§ significantly different than Group 3,

‖ significantly different than Group 4 (p<0.05, ANOVA, Kruskal-Wallis test, Bonferroni-Dunn test).

AL: clinical attachment loss, BOP: bleeding on probing, GI: gingival index, PI: plaque index, PISA: periodontal ınflamed surface area, PESA: periodontal epithelial surface area, PPD: periodontal pocket depth

**Figure 1:**
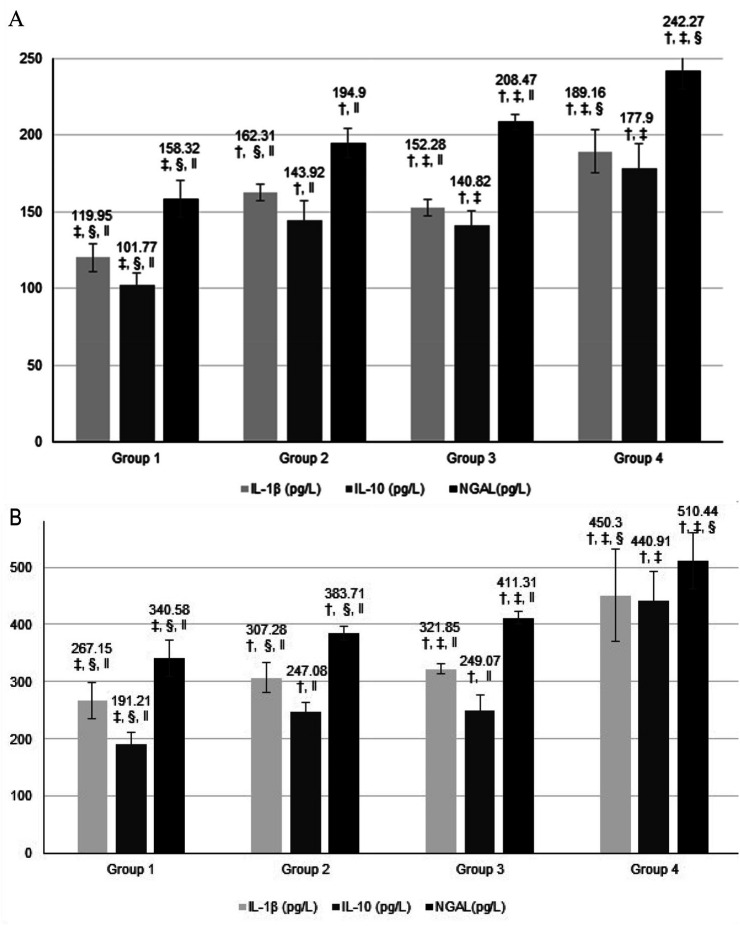
A: Salivary IL-1 β, IL-10, and NGAL levels, B: Serum IL-1 β, IL-10, and NGAL levels and comparisons between groups. †: significantly different than Group 1, ‡: significantly different than Group 2, §: significantly different than Group 3, ‖: significantly different than Group 4 (p<0.05, ANOVA and Tukey's Test).

Figure 1 shows salivary (Figure 1a) and serum (Figure 1b) IL-1β, IL-10 and NGAL levels and comparisons among groups. Salivary IL-1β levels were significantly higher in Group 2 than in Groups 3 and 1 (p=0.045, and p=0.000), but significantly lower than Group 4 (p=0.000). However, salivary IL-10 levels showed no statistically significant differences between Groups 2 and 3 (p=0.923), but were significantly lower in Group 1 when compared to Group 4 (p=0.000). Salivary NGAL levels increased significantly alongside disease progression from periodontally health to stage III periodontitis; all groups were significantly different from each other (p=0.000).

Serum IL-1β levels progressively increased from group to group, from periodontal health to periodontitis, and were significantly different among groups (p=0.000). Although serum IL-10 levels increased in the transition from periodontally health to periodontal compromised groups (p=0.000), Groups 2 and 3 presented no significant differences (p=0.450). Groups 2 and 3 had significantly lower IL-10 levels than Group 4 and higher levels than Group 1 (p=0.000). Serum NGAL levels increased significantly alongside disease progression, and all groups were significantly different from each other (p=0.000).

All clinical periodontal parameters were significantly and strongly correlated with other clinical periodontal, serum, and salivary parameters. All serum and salivary parameters were significantly and strongly correlated with all other serum and salivary parameters (p=0.000, data not shown, added as supplementary material).

## Discussion

This cross-sectional study aimed to investigate whether increased periodontitis severity, bleeding, and ulcerated periodontal inflamed surface area (PISA) increase NGAL levels in local (saliva) and systemic environments (serum). We also investigated whether gingivitis causes a systemic inflammatory burden as high as periodontitis. Our results verified and answered these study questions.

Sociodemographic parameters and characteristics regarding education level, monthly income, dental visit frequency, and oral hygiene habits were not significantly different among groups. The significant age difference between groups (lower age in Group 1) could be deemed as a limitation. However, to the best of our knowledge, there are no studies in the literature reporting that age could affect serum and salivary IL-1β, IL-10, and NGAL levels. In turn, the available knowledge on the effects of ageing in immunity^31^ led us to understand that age differences between groups cause no impact on the investigated parameters, considering the lack of individuals older than 65 years in our study population.

We found significantly higher body mass index (BMI) values in stage III periodontitis group than in healthy group, although similar to other groups. Our study groups presented mean BMI below obesity limit, suggesting that obesity level does not affect our study population periodontal status and disease severity. BMI values were not significantly correlated with salivary and serum parameters (data not shown). Meta-analysis and systematic reviews state that overweight, obesity, weight gain, and increased waist circumference may represent risk factors for the development of periodontitis or worsening of periodontal condition.^32^^,^^33^ Corroborating our results, Syrjäläinen, et al.^34^ (2019) reported that salivary cytokine concentrations were affected by periodontal severity rather than by obesity. Satpathy, et al.^35^ (2015) suggested that serum IL-1β levels were higher in periodontitis patients with abdominal obesity. Our study did not employ waist circumference and waist to hip ratio, only BMI values; yet, patients with BMI higher than 29 kg/m^2^ were not considered cases of abdominal obesity. Arboleda, et al.^36^ (2019) conducted a review to summarize epidemiological studies, and suggested that the association between obesity and periodontitis may be overestimated due to the different definitions of periodontitis cases adopted by their evaluated studies. In our study, BMI difference between healthy and moderate-severe periodontitis group could be considered a limitation, and our results should be evaluated with caution.

Among the studied clinical periodontal parameters, the periodontally healthy group presented the lowest plaque index (PI) and gingival index (GI) values, while stage III periodontitis patients (Group 4) presented the highest values. Oral hygiene habits influenced disease presence and/or severity in our study population.^37^ Groups 3 and 4 showed the highest bleeding on probing (BOP) values. Plaque and edema presence was similar between the two groups (gingivitis and stage I periodontitis). Yet, tissue damage was more evident in the stage I periodontitis (Group 3) than in gingivitis patients (Group 2), indicating that periodontal disease progression is related to immune response to biofilm.^38^ Bleeding, which indicates ulceration of pocket epithelium, is an important finding that links periodontal diseases to systemic diseases. PISA,^3^ a continuous periodontal variable in periodontal medicine studies used to calculate surface area bleeding and obtained by BOP, periodontal probing depth (PPD) and attachment loss (AL) values, was significantly different among groups. Groups with gingivitis (Group 2) and stage I periodontitis (Group 3) presented overlapping values.

Despite the belief that PISA may help classify the degree of periodontal disease, PISA threshold values are yet to be determined. In our study, PISA values were consistent with the current classification for periodontal diseases^22^ (as well as with the former CDC-AAP classification),^24^^,^^25^ and increased with periodontal disease presence and increased severity. When compared to the other studies,^4^^-^^6^ the higher PISA values reported in our study may be explained by the periodontitis generalized involvement of our groups. Another important finding suggests that gingivitis can cause as much systemic inflammatory burden as stage I periodontitis: the overlap in PISA values and the positive strong correlations between PISA and pro- and anti-inflammatory biomarker levels in gingivitis and stage I periodontitis groups. To the best of our knowledge, this is the first study to investigate serum and salivary NGAL levels considering different periodontal diseases and severity. We found all periodontal parameters and PISA values to proportionally increase with increased disease severity and NGAL levels.

Periodontal disease progresses if the balance between pro- and anti-inflammatory cytokines in periodontal tissues is disturbed in favor of pro-inflammatory cytokines.^7^ We investigated IL-1β, a key pro-inflammatory cytokine in tissue damage. Salminen, et al.^39^ (2014) showed that salivary IL-1β levels were critical for periodontal disease progression. In our study, serum IL-1β levels progressively increased from healthy (Group 1) to stage III periodontitis (Group 4) and salivary IL-1β levels were higher in gingivitis group than in stage I periodontitis group. This finding confirms that IL-1β is an important cytokine in the transition from gingivitis to periodontitis, corroborating results reported in the literature.

We also investigated another important anti-inflammatory cytokine, the IL-10. Information on IL-10 levels reported in the literature are still conflicting: whereas Andrukhov, et al.^40^ (2011) reported that serum IL-10, TNF-α, and IFN-α levels were higher in individuals with periodontitis than in healthy individuals, Teles, et al.^41^ (2009) reported that saliva IL-10 levels were lower in periodontitis patients than in healthy individuals and salivary IL-1β levels were higher in periodontitis patients than in healthy individuals. In our study, stage III periodontitis (Group 4) presented the highest values of both serum and salivary IL-10 levels, corroborating Teles, et al.^41^ (2009), while the periodontally healthy group (Group 1) presented the lowest values. We found no statistically significant difference between gingivitis and stage I periodontitis, in line with our study hypothesis. This may suggest that periodontal disease may progress more rapid in individuals with low anti-inflammatory capacity, and that the immune system of individuals with gingivitis is as active those with in stage I periodontitis.

A study reported circulating NGAL to be regulated by IL-1β in systemic inflammatory conditions.^16^ IL-1β was reported to be the major inductive cytokine of NGAL by NF-kB activation in an experimental acute-on-chronic liver failure model.^16^ The positive strong correlations between serum and salivary NGAL and serum and salivary IL-1β found in our study support these studies. Increased serum and salivary IL-1β levels induced the increase in serum and salivary NGAL levels from healthy to severe periodontal disease.

By investigating the association between NGAL and IL-10, a study found that the inhibition of NGAL decreased IL-10 expression by macrophages and the pro-proliferative and anti-inflammatory NGAL role in a renal ischemia-reperfusion model.^18^ The role of NGAL in periodontal disease pathogenesis, as regulated by IL-1β and IL-10, could be conjectured within the limitations of this study, as supported by the increased serum levels progressing from health to disease. In our study, serum and salivary NGAL levels were statistically different among the study groups by progressively increasing with increased severity and PISA. Serum and salivary NGAL showed a significant and strong positive correlation with periodontal clinical parameters and PISA, suggesting that NGAL plays an increasing role in cases of periodontitis with exacerbated inflammation and increased ulcerated bleeding surface area.

Our study poses several limitations. First, its cross-sectional design does not enable an analysis of NGAL in the pathogenesis of periodontal disease, which requires future randomized case-control studies investigating the cut-off values of serum and salivary NGAL levels in periodontally healthy and compromised patients, and employing interventional approaches (comparing NGAL levels before and after non-surgical periodontal therapy). Secondly, the presence of obese patients in periodontally compromised groups (although not significant among groups) might have affected salivary and serum NGAL levels. As fat distribution (determined by waist/hip ratio or waist circumference) was reported to be associated with periodontitis rather than higher BMI,^42^ the lack of such measurements could pose a limitation to observe clear associations between the investigated parameters.

## Conclusion

Despite the limitations of our study, NGAL seems to play a role in the pathogenesis of periodontal disease, as its increasing level is associated with the presence and severity of the disease. This finding is supported by the significant and strong correlations between NGAL and clinical parameters, as well as serum and salivary IL-1β and IL-10 levels.
